# A historical and political epistemology of microbes

**DOI:** 10.1111/1600-0498.12300

**Published:** 2020-07-12

**Authors:** Flavio D'Abramo, Sybille Neumeyer

**Affiliations:** ^1^ Max Planck Institute for the History of Science Berlin Germany; ^2^ Indipendent Artist Berlin Germany

**Keywords:** antibiotic resistance, health diplomacy, history of microbiology, microbial ecology, microbial epistemology, pandemics

## Abstract

This article traces the historical co‐evolution of microbiology, bacteriology, and virology, framed within industrial and agricultural contexts, as well as their role in colonial and national history between the end of the 19th century and the first decades of the 20th century. The epistemology of germ theory, coupled with the economic interests of European colonies, has shaped the understanding of human‐microbial relationships in a reductionist way. We explore a brief history of the medical and biological sciences, focusing on microbes and the difficulty of implementing germ theory outside of biology laboratories. Furthermore, we highlight the work of Lynn Margulis, who conceptualized microbes within their ecological contexts. Such research shows the active role microbes play in handling life‐sustaining biological and biochemical processes. We outline how the industrial and technological advancements of the last two centuries not only impacted almost all human societies, but also changed the world on microbial, biological, and geological levels. The narration of these histories is a complex task, and depends on how national, international, and intergovernmental institutions (such as the World Health Organization) conceive of the selective environmental pressures exerted by industry and biotechnological companies.

Our historical analysis is an example of microbial epistemology, which is the entangled interactions between economy, science, and politics that contribute to the interpretation and usage of changing knowledge about microbes.^1^ This article offers a short, non‐exhaustive history of the science and culture surrounding microbiology, bacteriology, and virology to trace the rise of predominant systems of knowledge that have shaped the modern‐day understanding of human‐microbial relationships.

Late 19th‐century scientific analysis and intervention on the microbial world fostered industrial production. Koch, Pasteur, and other scientists in Europe and its colonies solved, for *some* humans, problems such as food shortages and epidemics. Within most of these scientific inquiries, microbes were viewed as pathogenic agents—that is, powerful actors that caused crippling disease for crops, livestock, and humans.^2^ Outside of biomedical settings, though, scientists such as Margulis and Winogradsky have conceptualized microbes within their ecological contexts to show how crucial they are in sustaining the most basic biological and biochemical processes of planet earth. In the last century, the analysis of microbes was repeatedly polarized, portraying them either as enemies to fight or as life‐sustaining elements. Despite the current pandemic, we posit that the latter position more usefully captures the “nature” of microbes amid environmental and social contexts. We argue that an appraisal of ecological models of microbes allows us to formulate a *longue durée* examination of national, international, and intergovernmental institutions that function as mediators between both science and politics as well as science and society.

## 1 THE INDUSTRIAL INCEPTION OF MICROBIAL SCIENCES

Historians often cite the rise of germ theory as the beginning of the transformation of traditional medicine. Miasma theory and other beliefs that associated disease with locations and climates slowly transformed into modern biomedicine, which is based on observable, microscopic entities. Yet, the study of material, social, and environmental conditions that impact biomedicine is often ignored.^3^ The use of microscopes helped biologists develop the central arguments of germ theory, which was based on the knowledge that transmissible microorganisms can infect larger organisms and cause diseases.

However, viruses are fundamentally unlike bacteria. Microbiologist Louis Pasteur, bacteriologist Robert Koch, and other European pathologists studied bacteria via microscopes and cultivated them in the laboratory, yet viruses eluded these experimental apparatuses. The tobacco mosaic virus, the first virus ever to be identified, was discovered by plant pathologists in an attempt to counter harvest losses in plantations.^4^ In 1886, the German bacteriologist Adolf Meyer named it the “tobacco mosaic disease.” Dimitri Iosifovich Ivanovsky isolated its cause in 1892 by examining the sap of infected leaves. He declared that the infectious agent was able to pass through the ceramic sterilizing filters originally used to catch bacteria, and its filtrate could spread the disease to other plants.^5^ Likewise, Friedrich Loeffler and Paul Frosch, both working at the Koch Institute for Infection Diseases in Berlin, investigated foot‐and‐mouth disease—a disease that caused substantial financial losses especially related to milk production and cattle breeding.^6^ They injected healthy calves with the virus and discovered that the filtered lymph fluid of animals with foot‐and‐mouth disease was able to infect other animals even when heavily diluted. The pathogen was therefore thought to replicate within the hosts.^7^

Building on the 1878 proposal of his colleague Charles‐Emmanuel Sédillot, Pasteur declared, “every virus is a microbe.”^8^ Before the advent of late modern microbiology, the term “virus” was used for a broad category of virulent, poisonous substances causing diseases. Pasteur's statement narrowed down the general concept of “virus” to refer to all small and alive microscopic agents such as bacteria, protozoa, or fungi.^9^ While many of these infectious agents could be observed and discovered under the magnifying lenses of the light microscope, some of them remained invisible. By the end of the 19th century, viruses were thus defined by traits of technical uncontrollability. Unlike bacteria, viruses could not be filtered with ceramic or porcelain, could not be cultivated in artificial, purified environments (such as in a Petri dish), and could not be observed through microscopes. A search for their characteristics and developmental patterns started.

A technological breakthrough came in 1931, when Ernst Ruska, with the help of his mentor Max Knoll, invented the electron microscope (*Übermikroskop*), which was used to produce the first image of a virus in 1938.^10^ This device helped scientists to better understand the peculiar and extremely small organisms, and “led to a thorough understanding of an entirely new world of pathogens.”^11^

Scientists began to understand how both bacteria and viruses could be managed. Although a disease pathogen could now be explained through the transmission of a specific microbe identified in a laboratory, what fundamentally caused an epidemic outbreak could also be understood within a broader ecological and social context. Take, for example, the French physician Eugène Jamot: in 1929, he discovered that the microorganism that causes sleeping sickness, the protozoa *Trypanosoma brucei*, is transmitted by the tsetse fly. He claimed that French colonization had fundamentally unbalanced African colonial ecosystems through the introduction of new crops, social displacement, human migration, and labour recruitment, and that it was these social and ecological changes that had given rise to the sleeping sickness epidemic.^12^ Regardless, he modelled sleeping sickness as a parasitic disease, not as the result of changing environments and interruptions of social structures. ^13^ In the urban environment of London, which suffered from cholera and typhoid throughout the 19th century, or in colonial environments such as British India, administrators took advantage of the biomedical knowledge to develop quick solutions, such as the use of therapeutics and vaccines, that would allow uninterrupted trade flows and labour regimes. Often biomedical solutions were prioritized over large‐scale infrastructural reforms—such as sewage, drainage, or slowing human labour migration—as methods to decrease disease transmission.^14^

The difficulties in implementing knowledge about microbes in social medicine, which is outside of laboratories and the processes of industrial production, were due to the economic, political, and colonial contexts in which these same scientific endeavours were funded.^15^ Germ theories were recognized as “resources for a military model of disease control … to identify the new *insurrectos* and place them within a complex of strategy.”^16^ Indeed, the study of bacteria and viruses was often developed to solve biological and biomedical problems that disrupted productive infrastructures of territories militarily occupied and governed by European colonizers. In other words, the scientific knowledge of bacteria and viruses developed by Koch, Pasteur, their colleagues, their students, and other biologists only interested medical and political institutions in respect to the diseases that microbes caused, regardless of the environmental and social contexts.

## 2 ENVIRONMENTAL AND EVOLUTIONARY EPISTEMOLOGIES OF MICROBES

Despite the predominantly clinical applications of germ theory, since the middle of the 19th century, the environmental, ecological dimension of microorganisms has also been highlighted as an elemental, creative biological force.

In contrast to cells and bacteria, which can be cultivated on Petri dishes, viruses cannot be grown on such media. The invention of alternative forms of microbiological culturing allowed microbes to be studied beyond the realm of disease. The Winogradsky column, developed by Sergej Winogradsky in the 1880s, introduced microorganisms as environmental, ecological forces that are capable of producing carbon dioxide and nitrogen, two elements necessary to life.^17^

Another study to examine microbes beyond their association with disease was conducted by Pasteur. In 1850, he began to investigate the productive role of bacteria in the agricultural processes of lactic and alcoholic fermentation.^18^ In 1885, to understand the digestion processes of livestock, Pasteur fed various farmyard animals with food artificially devoid of the most common microorganisms, to eventually state that life without microbes would be impossible.^19^ According to Robert Koch, the doubt as to whether microbes were merely pathologic arose during the Hamburg cholera epidemic in 1892, in which individuals carrying the pathogen appeared not to fall ill.^20^

The controversial 19th‐century clinical observations of asymptomatic carriers, coupled with the systematic study of the microscopic world in the laboratory, sparked a heated debate on the transformative dynamics of microbes and their relationships with their host organisms. Fixity of species was maintained by Linnaeans like Ferdinand Cohn, who structured the most successful taxonomy of microbes.^21^ He was countered by scholars, including Ernst Hallier and Carl von Nägeli at the end of the 19th century, who saw microorganisms as able to develop in different forms depending on environmental factors, such as nutrition.^22^ Since the beginning of his studies, and cautiously countering any Linnaean idea of microbial fixity, Pasteur had rejected any species‐specific characteristic of bacteria, and he maintained this belief as he developed experimental practices to modulate the virulence of these microorganisms.

In 1950, about a century after Pasteur's studies and following unvaried appreciation of environmental factors, it was shown that once viruses are stimulated by environmental stress, they emerge from dormancy and multiply inside the bacterium in which they live. They then erupt from the bacterium, kill the host, and spread copies of themselves, along with some of the bacterial genes of the host, into the environment.^23^ These copies then infect nearby bacteria, and the cycle begins again.

Underpinned by the molecular revolution, current definitions of a virus mostly describe them as:

a package of genetic information protected by a protein shell for delivery into a host cell to be expressed and replicated [which] eventually takes over the cell completely for its own replication and in the process may kill (lyse) the cell or, in the case of tumor viruses, can permanently alter the cell.^24^

Another common definition describes them as, “too small to self‐maintain … until they enter … a bacterial cell, the cell of an animal, or of another live organism.”^25^ As the virus is unable to reproduce on its own, it is often considered a mere mixture of chemicals.

Nowadays, omics technologies applied to phylogenetic analysis have shown that the universal common ancestor, as depicted in the tree of life, was bacterial.^26^ Bacteria and viruses are conceived as fundamental elements necessary to all forms of life and which, throughout more than 4,000 million years, have combined and transformed basic chemical and biochemical elements to give rise to complex forms of life. The genomes of all cells in all species could be the result of the intensive evolutionary activity of horizontal gene transfer carried out by microorganisms such as bacteria and viruses.^27^ The theory of symbiogenesis completes the study of horizontal gene transfer. Beyond the medical framework of bacteriology, symbiogenesis frames the understanding of microbial life as the necessary, elemental dynamic of all living phenomena.^28^ It is subversive of traditional conceptual frameworks: in biology, it challenges the boundaries of the organism; in politics, it challenges identity projects based on the erasure of diversity by instead highlighting parasitism as the foundational dynamic of community formation.^29^ The term symbiogenesis was made popular in the late 20th century by Lynn Margulis, who claimed that microorganisms are one of the major evolutionary forces in the origin of species as the endosymbiosis of bacteria is responsible for the creation of complex forms of life.^30^ Margulis theorized that eukaryotic cells evolved from a symbiosis of bacteria without nuclei that had previously lived independently. In her theory, both chloroplasts and mitochondria, which are organelles found in all plant and animal cells, respectively, evolved from once free‐living bacterial species.^31^ This serial endosymbiotic theory gave microbiology a new evolutionary dimension and helped explain the origin of cells with nuclei. Margulis's idea was, and remains, highly innovative: cells with nuclei, including all the cells in the human body, descend from bacteria that formed symbiotic relationships more than 2 billion years ago.

After decades of disregard in microbial research, there is a renewed interest today in bacteria and viruses as elemental subjects of biological, geological and atmospheric dynamics. The recent attention is shifting notions of microorganisms as threats to human and non‐human health by acknowledging their key roles in Earth systems.^32^ Viruses and bacteria facilitate the construction of genetic patterns that underlie all organisms. Together with other microbes, they co‐maintain environmental equilibria, which make them key actors in both evolutionary and ecological dynamics. Maureen O'Malley looks at viruses beyond their small‐scale interactions with hosts and their role as disease‐causing agents. She emphasizes that understanding viruses from an ecological perspective allows us to consider that they “should be conceptualized as ecological actors that are at least comparable and possibly equal to organismal actors.” She further argues that, despite ongoing disputes, “organismality does not matter.” Viral ecologies and epistemologies allow us to grasp “*that life may be best understood as the combined and often non‐linear interactions of biological agents not all of which are living things*.”^33^ Concerned about the misinterpretations of biological systems, Lynn Margulis traces the human‐virus relationship back within the evolutionary landscape to highlight the imbrication between animals, plants, and viruses: “Viruses are no more 'germs' or 'enemies' than are bacteria or human cells. Like bacterial symbionts, viruses are sources of evolutionary variation. Viruses today spread genes among bacteria and humans and other cells, as they always have.”^34^

## 3 GERM THEORIES WITHIN INTERNATIONAL HEALTH ORGANIZATIONS

Why has ecological knowledge about microorganisms developed over the last two centuries been put aside to instead foster public opinion of microbes as pathological enemies to fight? The disruption of international trade and labour by epidemics and pandemics over the course of the last two centuries has pushed diverse governments, physicians' organizations, and health offices to create international, functional, non‐political conferences and institutions. Meetings like the International Sanitary Conferences and institutions such as the Pan American Sanitary Bureau, the Office International d'Hygiène Publique, and the League of Nations Health Organization took shape during the 19th and 20th centuries to track and report on the spread of epidemics in the Americas and Europe.^35^ Since its inception in the 1950s, the World Health Organization, which belongs to the lineage of international health activities of the last two centuries, has been conceived as a non‐political way to overcome the competition among nation‐states that led to the First and Second World Wars.^36^ Its international coordination role has facilitated the overcoming of many epidemics and pandemics. Nevertheless, the WHO adopted tactics that shifted the war from the human realm to the natural sphere. Among its top priorities have always been battles against diseases and microorganisms through the use of three “magic bullets”: antibiotics, DDT, and vaccines to eradicate venereal diseases, malaria, and tuberculosis.^37^

Before World War II, Gerhard Domagk showed that sulphonamides could kill bacteria.^38^ During the war, penicillin was produced at an industrial scale, representing the first biotechnological endeavour in which the engineering of productive processes backed up drug manufacture. As a “magic” solution against infectious diseases, penicillin was soon produced at an industrial scale and saved the lives of many. A century of widespread industrial usage of antibiotics instigated a so‐called antibiotic resistance, aggravated in recent years to the point that some describe it as an epidemic.^39^ Even though antibiotic resistance has been observed for about a century in response to penicillin and other human‐made drugs and chemicals, the industrial production of antibiotics has increased annually as a result of expanding activities based on the extraction and refinement of coal and oil.^40^ Massive industrial spills of antibiotics into natural environments resulted in antibiotic resistant genes that are exchanged horizontally among bacteria and viruses—natural guests of all living beings. For these reasons, several regulatory agencies and international health organizations, such as the European Medicines Agency, the European Centre for Disease Prevention and Control, the Centers for Disease Control and Prevention, and the Food and Drug Administration are in a phase of lowering the tolerance of antibiotics use in both veterinary and human medicine. In addition to tracking and monitoring antibiotic resistance, these institutions focus on how industrial activities, through the global production and trade of food, propagate the material conditions, both social and ecological, needed for their very existence. As Heather Paxson notes, these microbiological materialisations “are the stuff of which politics can be made,” and are used to propagate diverse laws and regulations, such as stricter regulations for migrant workers and imported food commodities.^41^

International institutions use experiment‐based understandings of nature to combat public health issues with a militaristic approach, which can be traced directly back to European industrial expansion of the 19th and 20th centuries. This tactic, however, ignores the ecological knowledge we have of microorganisms. Promising alternatives to antibiotics, for instance, can be found in a century‐old branch of research focused on bacteriophages or phages, a class of viruses that can selectively degrade the membranes of bacteria and other microorganisms.^42^ This research field, which during the last decades could not compete with the financial efficiency of antibiotic production and trade, would now enhance our comprehension of the relationships between viruses and bacteria to benefit the health of all animals and plants.

Moreover, to fully appreciate the discourses on epidemics and pandemics, it is necessary to look at the use of viruses and bacteria made within national defence programs. Indeed, in recent decades commercial bioindustrial facilities in the USA have developed vaccines, laboratories, and other technologies that can be used for both civilian and military purposes.^43^ Over the past century and despite the international norms against the military use of poison and disease, countries such as the USA, Soviet Union, and Japan have developed and deployed microbes as part of national defence programs.^44^ Such actions raise significant questions as to the relationships between microbial epistemologies and the administrative activities of national, international, and intergovernmental institutions, such as the UN and the WHO. The lack of transparency surrounding classified microbial research hinders the evaluation of related risks and threats. In contrast, when virologic research is carried out for civil uses, it encourages scientists to express their doubts about the research and voice their distrust of both the national agencies and international institutions that authorize these highly dangerous endeavours.^45^ Given that in the past biological warfare research has been carried out under civilian cover, further research is required to analyse the interaction between virology, bacteriology, and biotechnology companies, and the national and international institutions in which civilian and military purposes overlap.^46^ As geopolitical equilibria shift, illness does not simply disrupt social and economic regimes; it can be implemented as a diplomatic or military mean of persuasion and offence.

## 4 CONCLUSIONS

This brief analysis of the scientific and social histories that underlie the complex conceptual transformations of microbes and viruses across the past two centuries shows how economic, technological, and political contexts have shaped aims, modalities, and cultural premises of microbiology and cognate fields of research like bacteriology and virology. These disciplines fundamentally analyse the relationships between humans, animals, plants, and microbes.

The concept of the “invisible enemy,” fuelled by germ theory and realized within programs of colonial biomedicine and national security, has been nurtured since the advent of microbiological, bacteriological, and virologic sciences in the 19th century. Portrayed so as to reinforce national and international economic agendas, this concept has uniquely cast microbes as harmful agents. A broader framework of environmental and ecological research that engages with the understanding of microbes as both elements necessary to all life processes and potentially pathogenic entities represents a compelling alternative to the “invisible enemy” framework.

The selective pressures exerted by humans, mainly through industrial and scientific endeavours, have revealed the global impact that anthropic activities can have on microorganisms—for instance, through the widespread use of antibiotics or the acceleration of climate change.^47^ We have shown how industrial activities have physically altered our environments on a microbiological level across the globe. We have also traced how fundamental conceptions of scientific research subjects—such as bacteria, viruses, and microorganisms—change synchronously with human societies and civilisations over time (for example, we showed how bacteria changed their genome to resist man‐made antibiotics). How historical and biological changes of living entities and of their relationships are narrated depends on how selective pressures exerted by human institutions, such as industry and science, are conceived.

Academic institutions are now faced with the challenge of reinterpreting the scientific methodologies that materially altered the equilibria of our environments in order to address today's global crises. The history of science enables to conceive microorganisms and the scientific inquiries around them within prominent, cultural, social, and political dimensions that directly impact the lives of all plants and animals on this planet. An inquiry into the historic, social, economic, scientific, and industrial contexts of the current COVID‐19 pandemic is therefore not only a matter of curiosity, but also a way to engage with the *longue durée* of the crisis.

The scientific methodologies underpinning decisions made by experts hide unstable historical, environmental, and political legacies that have fragmented agencies across wide geographical and temporal networks. To historically map the social and biological impact of scientific methodologies is a particularly complex task. On the one hand, it involves the understanding of scientific models that are able to describe and interact with a world made of diverse living and non‐living interacting entities. On the other hand, it involves the understanding of the aims and impact that these scientific models have, not only on our societies and within the national and international health institutions, but also on a material, biological level. A right approach to historical and political analyses needs to clearly see that national and international health institutions constantly mediate between the social and political dimensions of scientific knowledge.

## AUTHORS' CONTRIBUTIONS

FD conceived the research project. FD and SN conducted the historical research. FD and SN developed the main arguments of this article. FD and SN drafted the manuscript. FD and SN have read and approved the final manuscript.

## Footnotes

^1^ In this article we use the term “microbes” to refer to both microorganisms such as bacteria and viruses.

^2^ Cassini et al. (2019).

^3^ Cf. Lock & Nguyen (2018, Ch. 4).

^4^ The tobacco mosaic disease was first observed in intensely cultivated tobacco fields in early 19th‐century Colombia. Its main cause was linked to the lack of crop rotation, which depleted the soil of vital nutrients, and thus increased the plants' susceptibility to disease. In the USA in the late 1940s, it “caused an annual average loss of 40,000,000 pounds of tobacco, accounting for 2–3% of the crop.” Scholthof (2004, p. 18).

^5^ Creager (2002).

^6^ At the end of the 19th century, the foot‐and‐mouth disease caused annual losses of 100 million German marks; cf. Schmiedebach (1999, p. 17).

^7^ Creager (2002); Schmiedebach (1999).

^8^ Summers (2014, p. 26).

^9^ Summers (2014).

^10^ Almeida, Leppanen, Maasilta, & Sundberg (2018).

^11^ Kruger, Schneck, & Gelderblom (2000, p. 1716).

^12^ Dozon (1991).

^13^ Lock & Nguyen (2018, p. 83).

^14^ Focusing on the Indian subcontinent, Sheldon Watts highlights that cholera is “the disease that imperialism kept in being for 130 years at the cost of some 25 million Indian dead.” Watts (1999, p. 207).

^15^ Kreuder‐Sonnen (2016).

^16^ Anderson (1998, p. 524).

^17^ Grote (2017).

^18^ Mendelsohn (1998).

^19^ Pasteur (1885).

^20^ Kreuder‐Sonnen (2016).

^21^ In the 1700s, Linnaeus named the microscopic infusoria *Chaos infusoria* (Sapp, 2012, p. 745), a moniker continuously used until the early 20th century, which shows the chaotic results of sorting microorganisms based on their morphology and regardless of their environmental contexts.

^22^ Kreuder‐Sonnen (2016).

^23^ This was shown by Esther Lederberg; cf. Schindler (2018).

^24^ Taylor (2014, p. 24). The molecular revolution, in which microorganisms played a central role, happened throughout the 20th century; cf. Rheinberger (2008).

^25^ Margulis & Sagan (1995, pp. 23–24).

^26^ Hird (2009). Omics disciplines are based on high‐throughput, automatized technologies whose shared data is part of global networks of biological and biomedical research.

^27^ The processes of genetic conjugation, transduction, and specialized transduction are described as *horizontal gene transfer* or *lateral gene transfer*. They refer to mechanisms of genetic exchange that are particularly relevant for the tempo and mode of microbial genome evolution. Horizontal gene transfer shows that bacteria can change their role through fast leaps, from benign to pathogenic or from pathogenic to benign. For instance, bacteria can develop antibiotic resistance and spread this resistance at the global scale; cf. Gillings (2017), Landecker (2016) and Sapp (2012).

^28^ Bosch & Miller (2016); Gilbert, Bosch, & Ledon‐Rettig (2015).

^29^ van Loon (2000).

^30^ Margulis (1998). The term symbiogenesis was first formulated by Konstantin Merezhkowsky in his 1926 book *Symbiogenesis and the Origin of Species* for the synthesis of new organisms by symbiosis. In 1927, Ivan Wallin proposed in *Symbionticism and the Origins of Species* that bacteria represent the fundamental cause of speciation; cf. Sapp (2005).

^31^ The endosymbiotic theory of Margulis is based on the findings of Wilhelm Schimper; cf. Sapp (2005).

^32^ Margulis (1998); Sagan & Margulis (1988).

^33^ O'Malley (2016, pp. 71, 77, 78).

^34^ Margulis (1998, p. 68).

^35^ Staples (2006).

^36^ Farley (2009).

^37^ During the first years of the WHO, social medicine was an important component of international analysis and programs that was substituted for the more pragmatic and market‐oriented takes of malariologists; cf. Farley (2009, Ch. 7).

^38^ Kreuder‐Sonnen (2016).

^39^ Cassini et al. (2019).

^40^ Landecker (2019a; 2019b). Antibiotics such as sulphonamides are derived from coal tar, which is a by‐product of industrial processes fuelled by coal. Moreover, there is scientific evidence that products and by‐products spilled out from petrochemical and industrial plants contribute in the development and spread of antibiotic‐resistant bacteria; cf. D'Abramo (2020).

^41^ Paxson (2019, p. 662).

^42^ Moelling, Broecker, & Willy (2018). In early 20th‐century epidemiology, phages were utilized to identify and differentiate bacterial strains; cf. Kirchhelle (2019). Today, phage therapy is conceived of as the most viable solution to the problems caused by bacterial resistance and other medical threats to human health, livestock, and crops; cf. Taylor (2014).

^43^ Tucker (2002).

^44^ The national, offensive use of bacteria and diseases in general overlaps with the birth of nation‐states; cf. Robinson (1971). Biological warfare programs of the Soviet Union started in 1928, and involved the production of bacteria and viruses to hit humans, livestock, and crops, as well as the development of vaccines and antibiotics; cf. Bozheyeva, Kunakbayev, & Yeleukenov (1999). The USA started its programs of biological warfare in 1941 through its Chemical Warfare Service, underpinned by the pharmaceutical firm Merck and Company, and these resembled the biological warfare activities of the Soviet Union; cf. Tucker (2002).

^45^ Butler (2015); Inglesby (2018); Koblentz (2010; 2017; 2020); Taylor (2014).

^46^ In 1970, the Soviet authorities began creating the biological warfare research complex named *Biopreparat*, formally run by civilian authorities and involving a large number of civilian biotechnological specialists and enterprises; cf. Bozheyeva et al. (1999).

^47^ Cavicchioli et al. (2019).
